# From Disease and Patient Heterogeneity to Precision Medicine in Type 1 Diabetes

**DOI:** 10.3389/fmed.2022.932086

**Published:** 2022-07-12

**Authors:** Nicoline H. M. den Hollander, Bart O. Roep

**Affiliations:** ^1^Department of Internal Medicine, Leiden University Medical Center, Leiden, Netherlands; ^2^Graduate School, Utrecht University, Utrecht, Netherlands

**Keywords:** autoimmune disease (AD), disease heterogeneity, disease endotypes, islet autoimmunity, genetic risk score, immune intervention therapy, type 1 diabetes immunopathogenesis

## Abstract

Type 1 diabetes (T1D) remains a devastating disease that requires much effort to control. Life-long daily insulin injections or an insulin pump are required to avoid severe complications. With many factors contributing to disease onset, T1D is a complex disease to cure. In this review, the risk factors, pathophysiology and defect pathways are discussed. Results from (pre)clinical studies are highlighted that explore restoration of insulin production and reduction of autoimmunity. It has become clear that treatment responsiveness depends on certain pathophysiological or genetic characteristics that differ between patients. For instance, age at disease manifestation associated with efficacy of immune intervention therapies, such as depleting islet-specific effector T cells or memory B cells and increasing immune regulation. The new challenge is to determine in whom to apply which intervention strategy. Within patients with high rates of insulitis in early T1D onset, therapy depleting T cells or targeting B lymphocytes may have a benefit, whereas slow progressing T1D in adults may be better served with more sophisticated, precise and specific disease modifying therapies. Genetic barcoding and immune profiling may help determining from which new T1D endotypes patients suffer. Furthermore, progressed T1D needs replenishment of insulin production besides autoimmunity reversal, as too many beta cells are already lost or defect. Recurrent islet autoimmunity and allograft rejection or necrosis seem to be the most challenging obstacles. Since beta cells are highly immunogenic under stress, treatment might be more effective with stress reducing agents such as glucagon-like peptide 1 (GLP-1) analogs. Moreover, genetic editing by CRISPR-Cas9 allows to create hypoimmunogenic beta cells with modified human leukocyte antigen (HLA) expression that secrete immune regulating molecules. Given the differences in T1D between patients, stratification of endotypes in clinical trials seems essential for precision medicines and clinical decision making.

## Introduction

Type 1 diabetes (T1D) is an auto-immune disease that causes insulin deficiency, affecting over 14 million patients worldwide. This results in blood glucose levels that are too high (hyperglycaemia) or too low (hypoglycaemia), which lead to frequent occurrence of both physical and mental difficulties: extreme thirst and hunger, muscle weakness and spasm, blurred vision, fainting, nausea, irritability, and poor concentration (1).

Patients require several insulin injections daily or an insulin pump to maintain healthy blood glucose levels. However, despite intensive monitoring and treatment most patients do not reach the guideline of a desired HbA1c for diabetics (<48 mmol/mol), let alone a healthy hemoglobin A1c (HbA1c) in general (<42 mmol/mol) (2). These guidelines are established to assure minimal risk of developing comorbidities later in life that result from poor glucose control (e.g., cardiovascular disease, damage to nerves, eyes, kidney, and feet). The mandatory effort to control blood glucose levels is very high, yet this dedication does not ascertain in-range glucose levels. Unexpected highs and lows are responsible for an average of an hour less sleep per night compared to the healthy population and an estimated 10 years shorter lifespan (2, 3). Furthermore, the overall psychological burden increases T1D patients' risk for depression. Treating insulin shortage is treating the symptoms of the disease, but not its cause; insulin is not a cure.

It has long been thought that T1D was caused by impaired antigen tolerance due to thymic dysfunction, as this is the case for several other autoimmune diseases (e.g., Myasthenia Graves, Addison, and severe combined immunodeficiency). However, recent research shows that healthy individuals also have CD8+ T cells that are specific for beta-cell proteins. Therefore, the mechanism of disease development remains elusive. Patients typically pass several physiological hallmarks before the symptoms of insulin deficiency start to show. Until today, it is still a topic of debate in which order this cascade of events takes place and how they affect disease progression. Also, many genetic and pathophysiological differences exist between patients. Altogether, it shows that T1D is a complex disease that requires more attention to ultimately cure. Most recently, the appreciation is growing that different etiopathogeneses may exist that cause disease heterogeneity, adding to the complexity and challenge to understand T1D and translate this understanding into disease intervention strategies.

T1D is complex in a sense that many factors seem to contribute to its onset. Several gene variants yield a higher risk, but environmental factors are also important. This idea arises from observational studies in which identical twins—with the same genetic background—may still have different disease outcome. Also, unrelated individuals that carry the same susceptible genes, but who live in different areas with different lifestyles, would have other risk. The incidence of T1D amongst young children increases too rapidly (2–3% annually) to be only caused by genetics (3). This suggests that certain environmental factors are needed to trigger the immune system and progress to clinical diabetes. Obesity, for example, causes chronic elevation of inflammatory cytokines, insulin resistance and metabolic and islet stress. Another hypothesis is that enteroviruses, which appear to be found in pancreatic tissue of some patients with T1D, induce HLA class 1 overexpression and CD8 recruitment (4, 5).

T1D has been proposed to undergo several stages of progression: (1) single islet autoantibody (Ab); (2) multiple islet Abs; (3) impaired glucose tolerance; (4) clinical diabetes (6). A single islet Ab may already be present before the age of 2, but this does not perse mean T1D development (stage 1). On the other hand, late-onset T1D is often accompanied by only one islet Ab. Also, the rate of progression differs between patients: some develop clinical diabetes within 1 year, some after 10 years or not at all, with rapid progression typically occurring in younger cases (7). However, if two or more antibodies are present (stage 2), further progression toward T1D is almost inevitable (84% risk), at least in the pediatric population (3).

## Disease Heterogeneity

**Figure 1:**
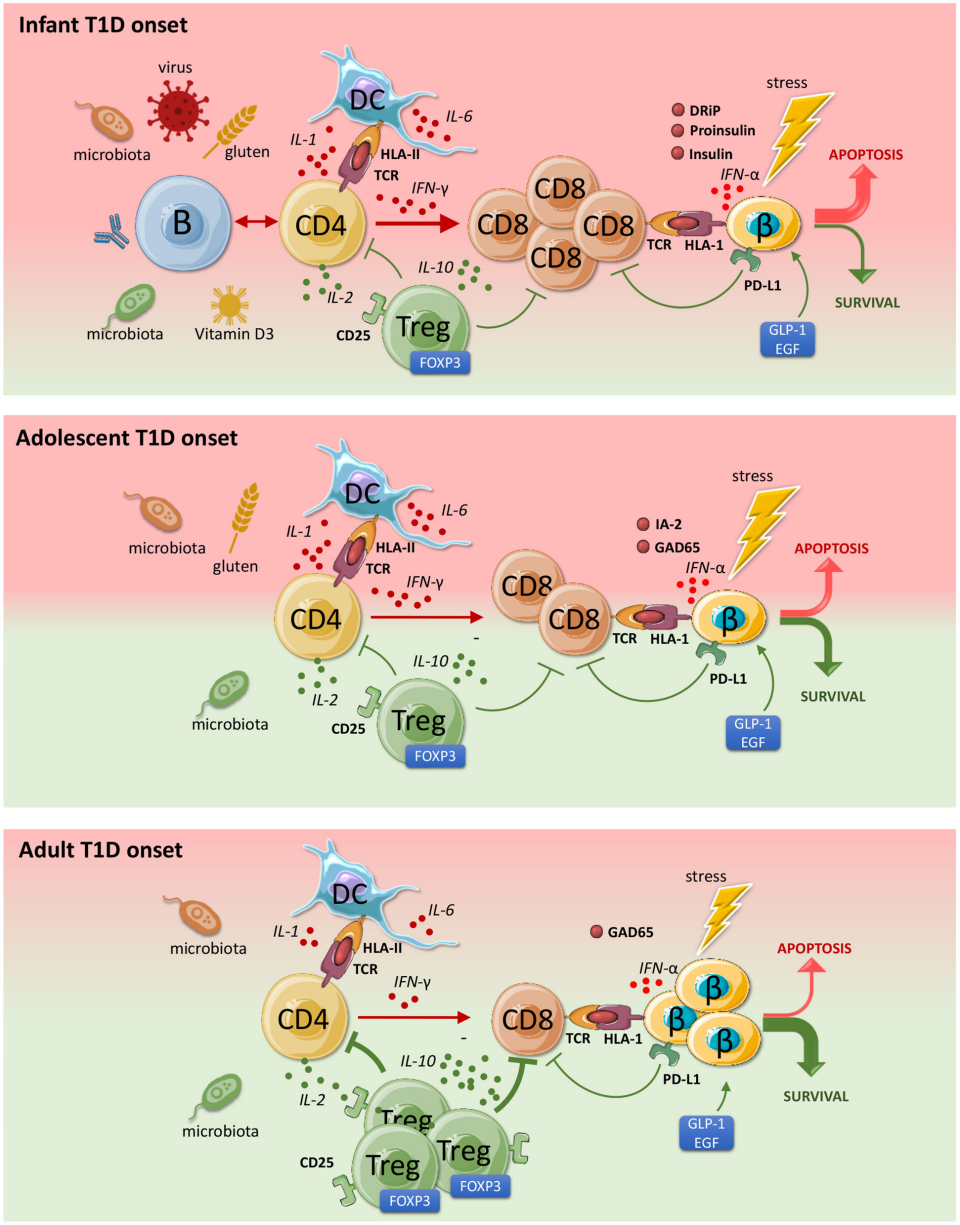
Pathophysiology and endotypes of type 1 diabetes (T1D). Both beta-cells and the immune system can provoke and diminish autoimmunity and beta-cell destruction. The contribution of the major cell types in disease progression differs depending on the age of disease onset. Green indicates a protective role and red points to a progressive role toward T1D development. The immune imbalance is greatest in patients developing T1D at younger ages, where the pathology is more acute and severe. With age, the degree of autoimmunity and the rate of beta-cell loss declines. CD4 T cells are activated by islet autoantigens that vary between disease endotypes. IL-2 differentially stimulates CD8+ effector T-cells as well regulatory T cells (low dose IL-2). Tregs protect against beta cell destruction. IL-6 stimulates inflammation and inhibits regulation. Activated CD8 T cells are triggered by IFN-alpha and IL-1 to attack beta cells. Due to stress, beta cells overexpress HLA Class 1 (HLA 1) and secrete IFN-alpha that provoke and attract CD8 cells. This destructive process may be inhibited by stress-reducing proteins GLP-1 and EGF. IFN-γ signaling stimulates PD-L1 expression and beta cell survival. This figure was partly generated using Servier Medical Art, provided by Servier, licensed under a Creative Commons Attribution 3.0 unported license.

The notion that T1D appears in different flavors depending of age at onset has generated much attention lately and the concept of disease endotypes was introduced to allow the design and assignment of different therapeutic intervention strategies depending on the disease endotype (precision medicine), as now is a common practice in high-income countries or communities for some diseases, including cancer (8–11). A pathological basis for this disease heterogeneity is still relatively weak, but the most compelling evidence derives from immunohistopathological studies of the lesion in the pancreatic islets showing differences in the rate and composition of the insulitic immune cells (Figure 1) (12). Patients diagnosed below the age of 7 (an arbitrary cut-off based on the available tissues) showed higher rates of inflammation dominated by CD8 T-cells but also featuring some B lymphocytes, whereas the rate and frequency of inflammation was less in children beyond the age of 12, while B lymphocytes were rare or absent in lesions at older onset (12). Strikingly, beta-cell features differed too: in cases with younger onset, insulin and its precursor proinsulin in the same subcellular compartments of the beta-cells whereas these molecules where not overlapping, suggesting that there may be intrinsic features of beta-cells too reflecting altered cell physiology, distress and immunogenicity that may contribute to a different and possibly faster disease progression than seen amongst patients with diagnosis at older ages (12).

In addition to differences in rate of disease progression and immunopathology associated with age, there are differences between patients at a given age related to, for instance, genetic background (HLA-DR3 and/or HLA-DR4; presence or absence of “protective” insulin gene variants) and ethnicity and ancestry (13). Commonly used Genetic Risk Scores designed to predict or diagnose T1D in patients of European descent did not deliver in populations of color (14).

Finally, the disease activity changes substantially with progression of disease, where insulitis appears most frequently in islets around diagnosis of T1D but is much reduced both in frequency and severity in prediabetic seropositive subjects or after diagnosis and restoration of glycaemic control with insulin therapy (12, 15, 16). This observation points out that the windows of therapeutic efficacy may depend on progression of disease, implying that some interventions specifically targeting insulitis may only be suitable or effective around the time of diagnosis, whereas other intervention strategies (for instance restoration of immune tolerance) would be better served not to be applied during the time around the medical emergency of diagnosis, insulitis and dysglycemia.

In this review we aim to find the most promising methods to cure T1D. This is investigated by sharing the latest knowledge and exploring different therapeutic approaches, covering both insulin production and auto-immunity reversal. Furthermore, the successful and less successful results from state-of-the-art studies are presented, highlighting the challenges in today's diabetes research and how to tackle these.

## Key Steps to Cure T1D

To cure a patient with clinically manifested T1D, two treatment goals need to be achieved: (1) reverse or suppress autoimmunity; and (2) restore insulin production. This treatment combination ensures that the new cells can provide insulin continuously, as they are no longer destroyed by the immune system.

## Suppress or Reverse Autoimmunity

Distinct cell types are associated with T1D development that may act in different patients (Figure 1). Both T cells and beta cells are involved in the pathogenesis, which likely induce disease onset in concert with dendritic cells and other antigen presenting cells (APCs), accompanied by B cells secreting islet-autoantibodies, which collectively cause beta cell destruction (8, 10, 17–21). The question remains which factor contributes to which, in whom and at which stage during disease progression. Thus, to answer this in the best possible way, researchers investigate pathophysiological changes and frequencies of immune cells in individuals that are at high-risk for T1D development (genetic or familiar susceptibility).

Over the past few years more evidence has been found regarding the imbalance between Tregs and Teff cells. The fact that non-diabetic individuals also produce islet-specific CD8 cells—although at lower levels—suggests that T1D has incorrect immune regulation by Tregs. Interestingly, genetically high-risk individuals are relatively protected when Treg levels are high (18). Furthermore, CD4+ T cells restricted by high-risk HLA class II alleles DR4-DQ8 and DR3-DQ2 present many islet-antigens such as insulin, islet-specific glucose-6-phosphatase catalytic subunit-related protein (IGRP), zinc transporter 8 (ZnT8), pre-proinsulin (PPI), while also secreting cytokines, which recruits and activates increasing amounts of CD8+ cells (19). Consequently, high levels of CD8+ cells that are reactive to glutamic acid decarboxylase 65 kDa (GAD65), IA-2, PPI, ZnT8, or IGRP or other islet autoantigen have been detected in islets of T1D patients (17). Tregs of patients appear impaired in their capacity to regulate effector T-cells (22). In addition, CD4+ cells in T1D patients are thought to be relatively resistant to Tregs (23). These pathogenic T cells are more responsive to IL-6, which promotes immune responses and further suppresses Tregs (24). These findings provide more insight into the imbalanced regulatory and effector immune system and how this causes greater immune responses in T1D.

Upon stressful circumstances, beta cells—together with resident macrophages—secrete IL-1 and express the chemokine CXCL10, which attracts more T cells toward the islets (25–28). Beta cells also produce TYK2 to undergo apoptosis, while further promoting inflammation by enhancing IFN-alpha signaling, upregulating HLA class 1 molecules and attracting CD8 T cells (29). Elevated levels of proinsulin are found right after food intake, which suggests that during this stage beta cells are still intact (stage 1–3), but there is a mechanistic defect in insulin processing that is responsible for glucose intolerance (12, 30). Recent findings suggest that after prolonged stress, beta cells secrete post-translationally modified proteins that behave like autoantigens: a phenomenon that is also seen in other autoimmune diseases (e.g., multiple sclerosis and rheumatoid arthritis) (10, 31–38).

Next to initial T cell and beta cell dysregulation, B cells may also contribute to disease progression, most likely in younger patients as high levels of pancreatic B lymphocytes were found in patients aged below 7 years old (16). Less IL-10 production by regulatory T and B cells is found in children with diabetes, which cytokine is found to play a protective role against T1D development (18). Also, gut bacteria stimulate IL-10 production and maternal antibiotics intake in non-obese diabetic (NOD) mice increases the risk of diabetes development in new-borns whereas low intrauterine gluten exposure and vitamin D3 supplements in human infants decrease the risk to develop T1D (39, 40). This further supports the idea that dysbiosis contributes to early T1D development (41–44). Curiously, fecal microbiome transplantation showed promise halting disease progression in newly diagnosed T1D patients (45).

Most immunotherapy-investigating trials are aimed at preventing further progression toward clinical diabetes by immune suppression (Table 1) (46). For the antigen-specific response, a T-cell receptor/CD3 complex is required to activate the T-cells upon recognition of this specific antigen. Teplizumab and Otilixizumab, both anti-CD3 mAbs, were found effective for delaying T1D in stage 1-3 diabetes patients (47–49). A similar drug, anti-thymocyte globulin (ATG), showed some effect at stage 2-3 (50). Both treatments caused less IFN-γ and TNF secretion by the disabled CD8 memory T cells, which is desired. However, this effect sustained for only 2 years and was soon taken over by CD8 expansion and disease progression. An ultimate reset of the immune system by autologous hematopoietic stem cell therapy induced complete remission in half of the treated newly diagnosed T1D patients that sometimes lasted for more than a decade (51, 52). Efficacy was observed in a subgroup of patients characterized by low CD8 T-cell autoimmunity to islets and superior immune regulation (51).

**Table 1:** Treatment strategies and pitfalls.

	**Mechanism (*Drug*)**	**Pitfall**
Autoimmunity	T cell deactivation; anti-CD3 (*teplizumab, ATG*)	CD8 expansion
	Co-stimulation blockade; anti-CTLA4/CD2 (*abatacept, alefacept*)	CD8 expansion when treatment stops
	Memory B cell blockade; anti-CD20 (*rituximab*)	Only effective in young patients
	Treg development; IL-2	CD8 expansion
	Antigen tolerance; oral insulin	Only effective in patients with IGT
Insulin	Progenitor cell differentiation *in vivo*	Unpredictable cell fates
	Porcine pancreas transplantation	Zoonosis, xenograft rejection
	iPSCs into liver	Necrosis, immunogenic environment
	iPSCs into pancreas	Redifferentiation and apoptosis
	Bio membrane-encapsulated iPSCs	Central necrosis, foreign body response

*Current approaches are shown that target autoimmunity (red) and insulin deficiency (blue). For each approach, the mechanism of action, drug (italic), and disadvantages (last column) are indicated*.

T cells are also controlled by binding of co-stimulatory ligands and co-inhibitory ligands. These ligands are perhaps more suitable targets to restore the balance toward immune regulation instead of attack (17, 46). Several immunotherapeutic agents have been developed that target these pathways. Promising drugs are abatacept inhibiting cytotoxic T-lymphocyte-associated protein 4 (CTLA4) (53–55) and alefacept targeting CD2 (56). Monthly treatment for 2 years with abatacept significantly reduced HbA1c levels compared to placebo, even after 1 year cessation. Alefacept improved C-peptide response and beta cell function, which improved HbA1c and reduced hypoglycaemia incidence. Alefacept also impairs CD8 function, confirming that T effector cells play a big role in disrupting beta cell function. Both drugs show good results in stage 3 diabetes, yet they require prolonged administration for sustained efficacy. Of note, disease was accelerated by abatacept in patients of color, pointing to both disease heterogeneity and the need for precision medicine.

Rituximab is an anti-CD20 antibody that depletes memory B cells and reduces auto-antigen presentation and attacks (57). This drug was only effective in a minority of stage 3 patients for a maximum duration of 1 year. Antibodies against insulin were less abundant, but antibodies against other beta cell derivatives remained the same. Interestingly, rituximab was more effective in younger patients, in whom pancreatic B cell infiltration is more profound. Individuals in which this treatment was less effective had increased levels of T-effector (Teff) cells, which supports the idea that treatment should target both B and T cells, at least in some cases (16, 58, 59). Efficacy of Rituximab to delay disease progression seemed limited to younger cases, whereas abatacept showed efficacy at older disease onset.

Furthermore, treatment with cytokines is being explored to enhance Treg development by increasing FOXP3 expression. Ultra-low interleukin-2 (IL-2) treatment reversed diabetes development in mice, however, in humans this effect was only temporary and led to expansion of CD8 cells. It seems that IL-2 is involved in both regulatory and Teff pathways, depending on other stimuli (60–62). Subsequently, a new treatment was developed that was more specific: stimulating CD25 and inhibiting CD122 receptor bonding (63).

Oral antigen administration effectively built tolerance and prevented development of diabetes in preclinical studies. This approach has been assessed in several clinical trials, showing some efficacy of oral insulin in a subgroup of patients but not all, and in particular in patients with higher titres of insulin autoantibodies (IAA) (17, 64, 65). Interestingly, more efficacy in delaying onset was also noted in patients with impaired glucose tolerance (IGT) than in patients without IGT who presented multiple autoantibodies. This suggests that such antigen exposure approaches are more effective in delaying further progression of stage 1 than as preventative treatment. Perhaps proinsulin would yield better outcome—especially in stage 0 patients—as this antigen is more immunogenic and its autoantibodies are present earlier during progression (66–68). Furthermore, preclinical studies suggest that low doses stimulate regulatory systems, while frequent or high doses cause depletion of effector cells (17, 69). Several other strategies to restore immune tolerance selectively without suppressing the immune system at large are currently explored that include injection with GAD65 (70, 71), proinsulin peptides in solution (72, 73) or presented by tolerogenic dendritic cells (74) and proinsulin DNA vaccination (17, 75).

## Preserve or Restore Insulin Production

Current successes in reversing autoimmunity are targeting up until stage 3 diabetes, in which a certain amount of beta cells is still intact. However, in clinical diabetes (stage 4) an estimated 90% of beta cells are already lost or non-functional (senescent) (76, 77). Hence, the source of insulin production needs to be replenished.

Theoretically, there are two strategies: (1) re-activation of senescent beta cells; or (2) pancreas, islet, beta cell or stem cell transplantation. The first focuses on beta cells that are still present, but are non-functional, due to distress or other often elusive factors. The internal biosynthesis machinery of beta cells has changed to a senescent (non-insulin producing or “hibernation”) status, which is conceivably a survival response, because active beta cells would be attacked by the immune system (10). Therefore, beta cell activation is only beneficial when combined with autoimmune reversal and immunotherapy. This approach requires external factors that switch the internal system, such as transcription factors or genetic modification with vectors that alter gene expression or treatment with beta-cell tissue factors such as epidermal growth factor (EGF) and GLP-1 that revitalize and activate beta-cell functions, respectively (78, 79). The possibility to differentiate progenitor cells in pancreatic tissue is also being explored (80). The major obstacle is that the differentiation process is quite unpredictable: multiple cell fates are induced, but their cues are largely unknown. Therefore, *ex vivo* differentiation and transplantation seem more promising, although the first clinical trial using this method is currently ongoing (NCT04786262).

The second approach uses cells from another source, which are already producing insulin (pancreas, islets, or beta cells) or are programmed to do so (stem cells). They should respond to glucose fluctuations adequately with the right amount and pace to prevent hypo and hyperglycaemia. To function as desired, they need to be placed within an environment that contains optimal circumstances and does not reject them.

Over the past decades, several insulin-producing transplants have been studied (80). Allogeneic pancreas transplantation is the only standard therapy suitable and available for a very limited number of T1D patients that leads to durable remission in the vast majority of recipients (81). Islet transplantation is still experimental and suffers from the lack of suitable organs and organ donors and the need for better immunotherapy (to prevent recurrent islet autoimmunity and allograft rejection) and improved and prolonged islet allograft survival and function (80).

Stem cells are another source of insulin production. Embryonic stem cells are the most pluripotent, but are taken from embryos, which is ethically questionable, and they need to match the recipients HLA type to avoid rejection. Induced pluripotent stem cells (iPSCs) are a more suitable source, as they are derived from patients' own stem cells (i.e., perfect HLA-match and ethically correct). Although clonal variability exists during the differentiation process of iPSCs, this source of insulin production is mostly investigated (82–84). Stem-cell derived beta-cells appear to be hypoimmunogenic and genetic engineering of stem cell-derived beta cells could further improve their resilience, protection, survival and function (85–87). Curiously, alpha-cells turned into insulin producing cells are also resistant to immune attack by islet specific autoreactive T-cells, pointing to either more resilience or reduced immunogenicity, or a combination thereof (88).

Now that several techniques for creating insulin-producing cells have been developed, the next caveat is finding the ideal implantation site and microenvironment for these cells. Implantation into subcutaneous tissue allows monitoring (observing abnormalities), implantation and removal, while internal tissue has more vascularization. Theoretically, the liver would be a logical choice, as glycogen is stored here. Additionally, the liver is the first organ through which absorbed glucose from the intestines passes. However, as described earlier, beta cells are stressed by high glucose levels, which makes them non-functional and self-destructive. Next to glyco- and lipotoxicity, the liver has a strong immunogenic environment, which increases the risk of damaging the implant (89). Furthermore, hepatic islet infusion showed little revascularization, which led to hypoxia and central necrosis. The fact that pancreatic islets receive five times higher blood flow, stresses the necessity of vascularization (90). However, researchers found that transplantation into the pancreas often leads to unwanted (de)differentiation, because of surrounding pancreatic differentiation cues (91). They also found that, the more stem cells differentiated (into beta cells), the more damage occurred. Only pluripotent cells (i.e., non-beta cells) were able to survive longer, but did not produce insulin (i.e., insufficient glucose-sensing). Implantation in alternative locations such as bone marrow or omentum proved less successful and required more donor tissue (92, 93).

Yoshihara et al. created human islet-like organoids (HILOs) that produce insulin and are responsive to glucose (94). Several differentiation steps were needed to induce beta cell-like characteristics, including non-canonical WNT signaling (maturation) and programmed death ligand-1 (PD-L1) overexpression (immune toleration). The optimal dose and frequency were evaluated using short multiple pulse stimulation (MPS) with interferon-γ (IFN-γ), inducing overexpression of PD-L1 without dedifferentiation or beta cell death. Transplantation of these cells led to sustained (>50 days) glucose homeostasis in both immune competent and humanized diabetic mice. Although IFN-γ is known to induce cancer in humans when overexpressed *in vivo*, this approach would solve this problem, as PD-L1 is expressed by *in vitro* IFN-γ stimulation prior to transplantation (94, 95).

Despite progenitor cells being well-programmed to produce insulin, it becomes clear that interaction with the surrounding tissue needs further optimalization. Isolated islets (e.g., cell cultures) go into necrosis easily when their surrounding is not providing natural stimuli that keep them active. The vascular network diminishes, which results in less nutrients and signaling molecules. Therefore, the use of hydrogels, matrices and other biomaterial-based devices could improve tissue circumstances. A promising method is the use of membranes that are permeable for nutrients but impermeable for immune cells (80). A phase 1 trial was effective but not sustained, as hypoxia and foreign body responses occurred. Currently, one phase-2 clinical trial investigates an encapsulated version (NCT04678557), while another trial uses bigger pores to reduce hypoxia combined with immunosuppressors (NCT03163511) (80).

Transplant recipients receive immunosuppressants, but these are not sufficient to reverse pre-existent B and T cell autoimmune reactions and often harm beta-cell differentiation and function (89, 96, 97). Immunosuppressive treatment usually consists of immediate specific drugs, followed by long term broad immunotherapy that blocks graft rejection. Low-dose systemic immunosuppressants reduce graft antigen rejection and prolong beta cell functioning (98, 99). Importantly, these drugs were selected to prevent rejection but are often uncapable to reverse pre-existent immune responses, such as islet autoimmunity, and therefore unable to prevent recurrence of autoimmune mediated beta-cell destruction (100–107).

## Challenges

In the twenty-first century many breakthrough solutions have been developed for a variety of diseases, however, T1D remains one of the few (severe) autoimmune diseases that cannot be cured yet by an approved therapy. Although much effort is put into different strategies, recent studies show that finding a cure for T1D is not as straightforward as earlier thought (17, 46). Therefore, it is important to map the currently faced obstacles to envision our next step.

First of all, diversity exists between T1D patients in several areas: genetics, onset age, progression rate, autoantibodies, and beta cell number and activity (8). This inter-subject variability makes it difficult to find one clear cause, as different factors contribute to the same clinical diagnosis. Therefore, the efficacy of treatment that reverses disease progression is highly variable among patients (Table 2) (11). This also explains why treatments often do not make it further than phase II/III clinical trials: their expected effect size is not met, because treatment has no effect in certain patient groups.

**Table 2:** Opportunities for precision medicine in type 1 diabetes.

**Intervention strategy**	**Preferred patient subgroup**	**Comment**	**References**
Oral insulin	Patients with high IAA titers	Confirmed in replication trial.	(65)
Diamyd (GAD65)	GADA positive patients	GADA negative have not been assessed.	(70)
	Patients carrying HLA-DR3?	Suggested by *post-hoc* analyses of multiple trials.	(108)
Tolerance induction with proinsulin peptide	Patients carrying HLA-DR4	Proinsulin peptide C19-A3 was eluded from HLA-DR4 molecules.	(18, 72, 74)
Abatacept	Adolescents	Replication warranted.	(53)
	European ancestry?	Disease acceleration in patients of color.	
Rituximab	Young patients	No efficacy achieved in adult patients; replication required. B-cells only present in insulitis in early onset T1D.	(57)
Islet transplantation	Patients lacking prior islet donor specific alloantibodies	A positive crossmatch consistently resulted acute allograft rejection.	(101, 105, 109)
	Patients lacking CD4 T-cell autoreactivity to GAD65 and IA2	None of patients responding to both GAD65 and IA-2 achieved insulin dependency, vs. >80% of patients not responding to either islet autoantigen; replicated in multiple studies.	(101, 105)
	HLA class I mismatch avoids recurrent islet autoimmunity by CD8 T-cells	Indirect recognition of islet epitopes by recipients' HLA class II cannot still occur.	(110)
	Patients lacking pre-existent thyroid peroxidase (TPO) autoantibodies	All patients with TPO antibodies developed Graves' disease following discontinuation of immune suppression after graft failure vs. none of the TPO-negative recipients.	(102)
Autologous hematopoietic stem cell transplantation	Patients with low rates of CD8 T-cell autoimmunity to islets	Patients with CD8 islet autoimmunity in the lower 50th percentile all reached complete disease remission at least up to 900 days after therapy at which time 85% of patients with high islet autoimmunity had relapsed.	(51)
Fecal microbiome transplantation	Autologous stool preferred over allogeneic stool?	Disease progression only halted in patients receiving their own stool; patients had not been randomized according to composition of microbiome or rate of islet autoimmunity. p.m.: Autologous microbiota have recolonization advantage.	(45)

Furthermore, beta cells modify their antigens under stressful circumstances. This mechanism of the immune system usually acts as surveillance against tumor cells that express neoepitopes (10). However, in this case the immune attack is targeted against beta cells, which might be an act of self-destruction; the new antigen is more immunogenic and has a higher binding affinity with cytotoxic T cells. Several studies show that post-translational modifications of beta cell epitopes are indeed strong triggers for T cell activation (10, 31–38). The neoepitope modification is thought to enhance binding affinity to high-risk HLA alleles compared to unmodified epitopes, which increases antigen presentation and recognition by T cells (111, 112). An alternative explanation for the cause of epitope modification is that the defective insulin protein is expressed under stress: an unwanted response that can be inhibited by verapamil, a drug that reduces oxidative stress and insulin-processing defects (113). Nevertheless, the formation of neoantigens complexifies the development of immunotherapy, as these antigens are highly variable between subjects. It is conceivable that immunotherapy alone will not suffice to reverse T1D durably. Combinations of beta-cell and immunotherapy may prevent continued provocations by distressed beta-cells to the immune system.

Another difficulty is translation from cell cultures to animal models to humans. Since beta cells are highly dependent on nutrients and oxygen (i.e., blood flow) and external stimuli, they function better *in vivo* than *in vitro*. On the other hand, they are more prone to re-differentiate *in vivo*, because of surrounding transcription factors and other signaling molecules. However, beta cells that are damaged or undergo necrosis secrete inflammatory cytokines that attract immune cells and cause damage to the well-functioning beta cells (10). In addition, a lack of good preclinical models of human T1D, the lack of access to the lesion in humans had delayed progress for decades. Since the access of diabetic pancreata through the establishment of the Network for Pancreatic Organ Donors (www.jdrfnpod.org), the insight in T1D has grown in a spectacular fashion, further underscoring the differences in autoimmune diabetes between species, but also pointing to the role of distressed islets preceding inflammation an revealing the relative paucity of inflammatory cells in human insulitis (with an striking lack of Tregs in the lesion), the low number of inflamed islets, the focal lesions, and the unexpectedly large number of insulin-positive islets seemingly unaffected at diagnosis (114).

Furthermore, transplanted cells are at risk for immune attacks and require additional immunosuppressive treatment. This becomes clear as transplanted islets slowly degrade within a few months (80, 89). Yet, broad immunosuppressants—such as cyclosporine used in the 80s—induce severe side effects such as kidney damage or infections (107, 115). Thereafter, research focused on development of more specific immunosuppressive drugs, such as IFN-γ to increase PD-L1 expression. Unfortunately, IFN-γ treatment also allows development of tumor cells as these are protected from immune surveillance as well (95).

A final hurdle is the fact that the control of T1D symptoms is relatively easy, increasing the bar for potentially risky therapeutic intervention strategies. In contrast to other autoimmune disease, where there is no alternative for immunotherapy, or cancer that would become lethal if untreated, insulin therapy deals with the hyperglycemia and diabetic complications only show years after diagnosis, masking the burning need for curative therapies. There is also a burning need to define efficacy of intervention therapy in T1D (116). At least seven types of clinical benefit were identified, varying from prevention of seroconversion in individuals at genetic risk, to prevention of epitope spreading, delay, reversal or prevention of T1D, to prevention of diabetic complication. Different immune intervention strategies may be amenable and effective in different stages of the disease and in different T1D patients (17, 46, 116).

## Novel Strategies and Future Perspective

**Figure 2:**
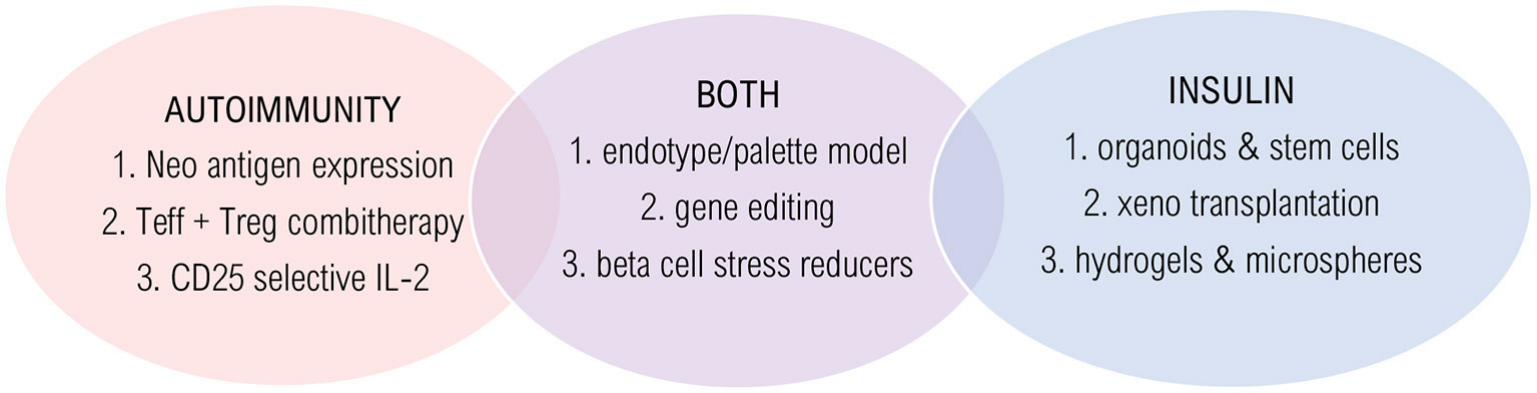
Innovative technologies and solutions. Promising strategies are shown that target autoimmunity (red), insulin deficiency (blue) or both (purple). Autoimmunity solutions: (1) Long-term and low-level antigen expression by AAV infection; (2) Combination therapy of effector T cell depletion followed by regulatory T cell stimulation; (3) Selective CD25-agonist/CD-122 antagonist. Insulin solutions: (1) Glucose sensing beta-cell like organoids that secrete sustained insulin by *ex vivo* WNT and IFN-γ signaling; (2) Humanized pancreas growth by patient-derived stem cell injection into pig blastocyst; (3) Hydrogels and TMTD-alginate microspheres to avoid rejection and necrosis. Solutions for both: (1) Trial stratification and treatment choice based on endotype; (2) Engineering hypoimmunogenic beta cells using CRISPR-Cas9; (3) Beta cell stress reducers to lower inflammation and increase beta cell function.

The post-translational modification of beta cell proteins (i.e., neoepitopes) explains why beta cells are suddenly not tolerated anymore, as the immune system should have been programmed to tolerate native epitopes by thymic selection. Therefore, research in antigen-based therapies should also focus on targeting neoantigens (Figure 2). Combining antigen with tolerogenic adjuvants, such as blocking T cell activation (e.g., anti-CD3) or co-stimulation (e.g., alefacept and abatacept) might limit the risk of severe side effects, which treatments were recently proven effective in mice and—to a certain extent—in humans. The major pitfall is that co-stimulation is also necessary for Tregs in humans, which might negatively affect the translation to the clinic. So, perhaps it would be beneficial to investigate a treatment regimen that depletes effector T cells, followed by Treg enhancement, as these two methods might interfere too much when administered simultaneously (17). Furthermore, as beta cell neoepitopes are a strong trigger for autoimmunity induction and are secreted under stress, agents that reduce beta cell stress (e.g., GLP-1 analogs, EGF, and Verapamil) could prevent autoimmune attacks (15, 117) (Figure 2).

Another evolving strategy is to induce Treg expansion by ectopic expression of antigen through antigen-encoding plasmid vaccines (75). In recently diagnosed patients, pro-insulin peptide injection increased IL-10 secretion and transcription factor forkhead box P3 (FOXP3) expression, and lowered beta cell-specific CD8 T cells and stress (72). Next, an intramuscular DNA vaccine to induce self-antigen expression—administered weekly for 12 weeks—induced significantly improved C-peptide levels until week 15 (75). Preclinical studies suggest that low doses of anti-CD3 combined with such antigen-delivery could build tolerance (118, 119). Given their long-term expression, adeno-associated virus (AAV) vaccines would be an interesting corrective gene delivery system for T1D, perhaps targeting multiple autoantigens, because only those CD8 T cells are reduced that are specific for the vector-expressed antigen (17, 75).

With the rapidly rising interest in the endotype concept, where subtypes of T1D could be based on pathobiological or functional mechanisms that cause T1D rather than the clinical characteristics, personalized intervention therapy comes in sight. Like asthma, treatments that target specific pathways are only effective in some patient groups, suggesting there are distinct subtypes of T1D with varying defective pathways that lead to a similar clinical result. It becomes clear that certain traits are linked, such as age, autoantibodies, and HLA-specific autoimmunity (120). For example, high-risk HLA allele DR4 is associated with early proinsulin autoantibodies. Similarly, high-risk allele DR3 is associated with GAD autoantibodies. These associations could form two distinct subtypes: proinsulin-DR4 and GAD-DR3 (8). Since drugs have very different efficacy based on low or high B cell titres, such as rituximab, B cell infiltration would also be a useful subtype.

Similarly, it would be useful to stratify clinical trials based on these variables, as described in the Palette model by McCarthy (121). He opts to cluster participants based on combinations of alleles, traits, and genes. Then, their clinical responsiveness to the investigational drug is mapped, which serves as a tool for clinical decision making: if the patient has similar characteristics to one distinct endotype, the treatment with highest benefit according to their endotype would be preferably administered. Stratification based on these endotypes would also lower variance, leading to higher power or significance, which would take precision drugs further toward phase 4 and to the market.

Lastly, whenever transplantation conditions are improved and the engrafted tissue is not rejected, there would still be a shortage of human donors, as they should be HLA-matched. Therefore, humanized pancreas growth in pigs is currently under investigation (122). This method blocks porcine pancreas growth by engineering their blastocyst and inserting patient-specific iPSCs. This technique was successful with mouse-derived stem cells, but still awaits confirmation in human trials. Another promising technique to tackle the shortage of (HLA matched) donors and graft rejection is to genetically modify beta cells using clustered regularly interspaced short palindromic repeats- associated protein 9 (CRISPR-Cas9) (70, 123). Insulin-producing beta cells were created that co-secrete IL-10, which protects them from immune attacks. Additionally, this technique could also knock-out endogenous HLA molecules and replace them with HLA-E or HLA-G to avoid attack of natural killer cells (124).

To conclude, treatment responsiveness depends on certain pathophysiological or genetic characteristics that are highly variable between patients. Therefore, future research may benefit from genome-wide association studies (GWAS) to gain insight into linked genes and characteristics, from which new T1D endotypes can be defined. Graft rejection or necrosis seem to be the most challenging obstacles, for which immune-tolerated and vascularized hydrogels and microspheres are promising strategies. Furthermore, stress reducing agents and CRISPR-Cas9 may preserve beta cell function. Since T1D remains complex and highly heterogenic, stratification of endotypes in clinical trials would help development of precision medicines and clinical decision making. Given the specular, ground-breaking and paradigm shifting new insight into the pathogenesis and diversity thereof of T1D, there is an urgent need to educate the different stakeholders (T1D patients and their loved ones, care providers, pharma, regulators and legislators) on realistic possibilities, expectation and outcomes that will differ between strategies and between patients (116). The future of T1D patients and the therapy of their disease has already changed for the better but there is still much to be gained.

## Author Contributions

All authors listed have made a substantial, direct, and intellectual contribution to the work and approved it for publication.

## Conflict of Interest

The authors declare that the research was conducted in the absence of any commercial or financial relationships that could be construed as a potential conflict of interest.

## Publisher's Note

All claims expressed in this article are solely those of the authors and do not necessarily represent those of their affiliated organizations, or those of the publisher, the editors and the reviewers. Any product that may be evaluated in this article, or claim that may be made by its manufacturer, is not guaranteed or endorsed by the publisher.
